# Betaine Inhibits Ferroptosis After Intracerebral Hemorrhage by Activating the Nrf2/HO-1 Pathway

**DOI:** 10.3390/antiox15010135

**Published:** 2026-01-21

**Authors:** Jie Chen, Xurui Lu, Sunqian Liu, Weiliang Hu, Xiaorong Zhou, Zhifeng Wang

**Affiliations:** 1 Department of Neurosurgery, Affiliated Hospital 2 of Nantong University/Southeast University Affiliated Nantong First People’s Hospital/Nantong First People’s Hospital, Nantong 226001, China; vecojay@163.com (J.C.); auroralu1999@163.com (X.L.); lsqvion@163.com (S.L.); huwl0824@163.com (W.H.); 2 Department of Immunology, Medical School, Nantong University, Nantong 226001, China

**Keywords:** ICH, ferroptosis, BET, Nrf2, HO-1, HT22, ROS

## Abstract

Intracerebral hemorrhage (ICH) is a type of stroke with high mortality and disability rates. The hemoglobin and iron ions released by ruptured red blood cells after ICH can induce programmed cell death characterized by lipid peroxide accumulation—a defining feature of ferroptosis—which is one of the key mechanisms for the occurrence and progression of secondary brain injury after ICH. Betaine (BET), a natural amino acid derivative, is known to be an antioxidant, but its protective effect and molecular mechanisms in ICH-induced ferroptosis have not been studied yet. In this study, we investigated the effect of BET intervention on ICH-induced ferroptosis and possible mechanisms in vitro and in vivo, and we evaluated the expression of ferroptosis and oxidative stress molecules through in vivo and in vitro experiments. We analyzed the distribution of nuclear factor E2-related factor 2 (Nrf2) and assessed neurobehavioral function, hematoma volume, and iron content in the brain tissue of mice with ICH. BET upregulates nuclear factor E2-related factor 2/heme oxygenase 1 (Nrf2/HO-1) signaling, reducing long-chain acyl-CoA synthetase 4 (ACSL4), reactive oxygen species (ROS), and malondialdehyde (MDA) while increasing glutathione (GSH) and glutathione peroxidase 4 (GPX4) levels. It also decreases brain iron accumulation, aids hematoma clearance, and protects against ferroptosis and oxidative damage post ICH. Inhibition of Nrf2 with ML385 diminishes BET’s neuroprotective effects, highlighting the pathway’s importance in BET’s mechanism of action. BET boosts antioxidant capacity via the Nrf2/HO-1 pathway; inhibits ferroptosis; reduces oxidative stress, brain edema, and iron accumulation post ICH; and aids hematoma clearance, offering neuroprotection.

## 1. Introduction

ICH accounts for only 28.8% of all strokes [1], but is considered a devastating disease due to high mortality and disability [2]. Clinically, the main treatment is surgical hematoma evacuation and life support, because there are no drugs to stop disease progression [3,4].

After ICH, erythrocyte lysis releases large amounts of free iron, and after entering cells, Fe^3+^ reduces to Fe^2+^, which directly induces ROS production by the Fenton reaction and promotes the conversion of polyunsaturated fatty acids (PUFAs) catalyzed by ACSL4 into phospholipid hydroperoxides (PLOOHs) [5,6]. This leads to a form of iron-dependent non-apoptotic cell death—ferroptosis—which is mainly characterized by lipid peroxidation and is one of the key pathological mechanisms of secondary brain injury after ICH [7]. GPX4, a key antioxidant enzyme, suppresses lipid peroxidation and metabolizes toxic lipid species [8,9]. Nrf2 upregulates the expression of downstream targets, including HO-1 and GPX4, and, in parallel, activates the transcription of glutamate–cysteine ligase (GCL) and glutathione synthetase (GS), thereby promoting GSH biosynthesis, which plays a pivotal role in antioxidant defense and ferroptosis inhibition [10,11].

BET (also known as trimethylglycine) (Section 3.1) is a naturally occurring “osmoprotectant” found in various plants and animals [12]. It is non-toxic, can cross the blood–brain barrier, and accumulates in the kidneys, liver, and brain after ingestion [12,13,14]. Studies show that BET has neuroprotective effects on the nervous system and delays cell aging and improves cognitive decline [15]. Moreover, BET has broad neuroprotective potential in models of neurodegenerative diseases and brain injuries such as epilepsy, Alzheimer’s disease, Parkinson’s disease, stroke, multiple sclerosis, and traumatic brain injury. In such models, BET reduces neuronal damage and relieves disease-related symptoms through its anti-inflammatory action and antioxidation, as well as by maintaining mitochondrial function stability [15,16,17].

The Nrf2/HO-1 axis is the “central command” of cells for ferroptosis. Following an ICH, Nrf2 breaks off Keap1, reaches the nucleus, activates transcription of antioxidant genes such as HO-1 and GPX4, and reduces the accumulation of lipid peroxidation products [18]. Previous studies have shown that BET can reduce oxidative stress-induced cardiovascular damage and pulmonary inflammation [19]. Supplementing with BET upregulates Nrf2 and HO-1 expression, increases GSH, and decreases the amount of MDA, increasing the antioxidant capacity of chickens [20]. In addition, studies have shown that BET can inhibit oxidative stress via this pathway, protecting the kidneys from adriamycin-induced damage [21].

BET has potential in neurodegenerative diseases. BET, together with boric acid (BA), corrected the imbalanced expression of ACSL4, GPX4, and transferrin receptor-1 protein (TfR1) in Alzheimer’s disease [22]. In addition, BET can alleviate ferroptosis by restoring the antioxidant defense system in granulosa cells [23], but no studies have reported whether BET inhibits ferroptosis after ICH via Nrf2/HO-1.

Therefore, in this study, we used a mouse collagenase-induced ICH model together with a hemin-stimulated HT22 cell in vitro ICH model to systematically test whether BET activates the Nrf2/HO-1 signaling pathway and inhibits ferroptosis, thereby exerting neuroprotective effects.

## 2. Materials and Methods

### 2.1. Animals

This study included 120 male C57BL/6 mice aged 8–9 weeks, obtained from the Experimental Animal Center of Nantong University. The animals were housed at a controlled temperature of 20–22 °C, with up to five mice per cage, under a standard 12-h light/dark cycle to maintain circadian rhythm. Food and water were provided adequately, and bedding was refreshed weekly to ensure optimal hygiene and comfort. All animal experiments followed the 3R principles, pre-experiments and statistical analysis minimized animal use, and improved modeling techniques and refined care reduced suffering and optimized procedures. Exclusion criteria: Mice that did not exhibit limb hemiplegia after the establishment of the ICH model were not included in the study. All experimental procedures were approved by the Animal Ethics Committee of Nantong University (S20250830-002, 30 August 2025), and conducted in strict accordance with the Guide for the Care and Use of Laboratory Animals published by the National Institutes of Health (NIH). The experimenters remained blinded to the experimental group assignments and drug intervention conditions throughout animal modeling, behavioral testing, and histological analysis.

### 2.2. ICH Models, Drugs, and Groups

Before the experiment started, all animals were deeply anesthetized by intraperitoneal injection of 400 mg/kg of 4% chloral hydrate solution, and once adequate anesthesia had been achieved, the heads of the mice were placed in a stereotaxic apparatus (RWD, Shenzhen, China). With a microinjection pump (Hamilton, Reno, NV, USA), 0.1 U of type IV collagenase was slowly injected into the right caudate nucleus of the mice to establish an acute ICH model. The injection target coordinates were 0.8 mm anterior to the bregma and 2.3 mm lateral to the midline, with a 3.5 mm depth [24]. After injection, the needle was held for 10 min to prevent backflow and then slowly removed, and the wound was sutured, after which the mouse was placed on a warming blanket. The sham group was treated similarly but 0.9% sodium chloride solution was used instead. BET (B2629, Sigma, St. Louis, MO, USA) was dissolved in 0.9% physiological saline and administered by intraperitoneal injection [25].

**Figure 1:**
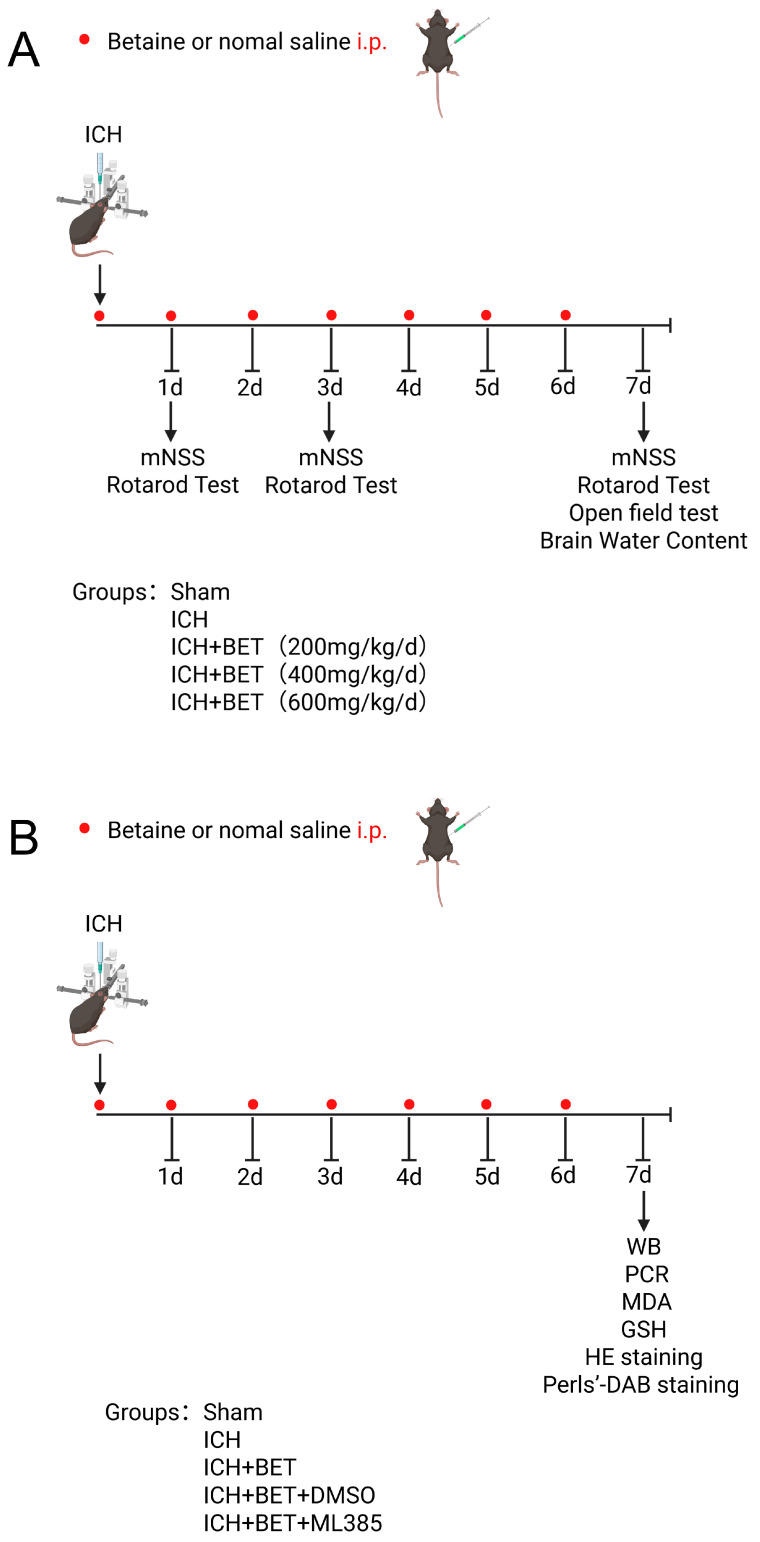
Flowchart of animal experiment design. Experiment 1 (**A**) and Experiment 2 (**B**).

In Experiment 1, the mice were randomly divided into the following groups: sham group (*n* = 6, 0.9% saline, i.p.), ICH group (*n* = 6, 0.9% saline, i.p.), and three ICH + BET groups (ICH + BET 200 mg/kg/d, *n* = 6; ICH + BET 400 mg/kg/d, *n* = 6; ICH + BET 600 mg/kg/d, *n* = 6). After ICH model induction, BET was administered intraperitoneally and for 7 days. Behavioral experiments and brain water content detection were conducted to obtain the optimal treatment dose and time (Figure 1A).

Experiment 2: The mice were randomly divided into the following groups: sham group (*n* = 18, 0.9% saline, i.p.), ICH group (*n* = 18, 0.9% saline, i.p.), ICH + BET group (*n* = 18), ICH + BET + DMSO group (*n* = 18), and ICH + BET + ML385 group (*n* = 18). ML385 (HY100523, MCE, Monmouth Junction, NJ, USA), a selective Nrf2 inhibitor, was administered at 30 mg/kg via 5% DMSO intraperitoneal injection 1 h before ICH modeling and once daily [26]. On the 7th day post modeling, the mice were sacrificed and brain tissue was collected (Figure 1B).

### 2.3. Brain Water Content

To evaluate the extent of brain edema, we used the wet weight/dry weight method. On the 7th day after the ICH model and drug intervention, mice were rapidly decapitated under deep anesthesia to extract the brain. The wet weight (WW) of these brain tissues was measured and recorded with an analytical balance (Ohaus, Parsippany, NJ, USA) at a precision of 0.1 mg. Subsequently, the weighed brain tissues were transferred to aluminum foil weighing boats and placed in a thermostatic oven at 100 °C for 24 h until a constant weight was achieved. After cooling to room temperature in a desiccator (approximately 30 min), these brain tissues were weighed again to obtain the dry weight (DW). The water content of the brain tissue was calculated using the following formula, Brain Water Content (%) = [(WW − DW)/WW × 100%], which accurately represents changes in brain tissue water content during the acute phase after ICH.

### 2.4. Open-Field Test

The open-field test evaluated the spontaneous locomotor behavior of the mice 7 days after ICH. The experimental device was a cube with side lengths of 50 cm, and its base was coated with a non-reflective white surface. Each mouse started at a standard starting point in the apparatus. A high-definition camera recorded the mouse behavior for 10 min. The movement trajectories, travel distances, and velocities of the mice were quantitatively analyzed using professional behavioral analysis software (Xinruan Instruments, Shanghai, China), enabling a comprehensive assessment of their motor function.

### 2.5. Rotarod Test

Rotarod tests were performed using an accelerating rotarod (Xinruan Instruments, Shanghai, Chian) on days 1, 3, and 7 post ICH to test the mice’s motor balance and coordination. In pre-training, the speed was 4 revolutions per minute and gradually increased to 40 revolutions per minute over 90 s. For the formal test, the mice were placed on the rotarod which accelerated uniformly from 0 to 40 revolutions per minute over 5 min. Latency to fall was recorded for each mouse.

### 2.6. Modified Neurological Severity Score (mNSS)

Neurological function was assessed using the modified neurological severity score (mNSS) after previous studies [27]. The mNSS criteria measure neurological function on a scale from 0 to 18, motor and sensory function, reflexes, and balance, where a maximum score of 18 is severe neurological function, while a minimum score of 0 is normal neurological function.

### 2.7. Cell Culture and In Vitro ICH Model

The HT22 (FH1027, Fuheng Biology, Xi’an, China) mouse hippocampal neuronal cell line was cultured in a 37 °C incubator with 5% CO_2_, using Dulbecco’s Modified Eagle Medium (DMEM) supplemented with 10% fetal bovine serum and 1% penicillin–streptomycin. Hemin (20 μM, HY19424, MCE, Monmouth Junction, NJ, USA) was used for 24 h to establish an in vitro ICH model [28,29], and HT22 cells were pretreated with varying concentrations of BET (B2629, Sigma, St. Louis, MO, USA) (0, 1.25, 2.5, 5, 10 mM) for 24 h prior to hemin stimulation.

### 2.8. Flow Cytometry

HT22 cells were seeded onto 6-well plates at a density of 1 × 10^6^ cells per well. Following the respective treatments for each group, the culture medium was aspirated and cells were gently washed with PBS to remove residual medium. Subsequently, a pre-diluted DCFH-DA (D6470, Solarbio, Beijing, China) probe was added to each well and incubated in the dark at 37 °C for 30 min to detect intracellular ROS levels. After incubation, unbound probes were thoroughly removed by washing twice with PBS. The cells were then trypsinized, collected via centrifugation, and resuspended in PBS. The resulting cell suspensions were transferred to flow cytometry tubes and analyzed using a flow cytometer (BD Biosciences, San Jose, CA, USA) with excitation at 488 nm and emission detection at 525 nm. Fluorescence intensities were quantified using flow cytometry analysis software to assess and compare ROS levels across treatment groups.

### 2.9. Determination of Malondialdehyde (MDA)

According to the kit instructions, brain tissues or cells were homogenized thoroughly in ice-cold RIPA buffer and centrifuged at 12,000× *g* for 15 min at 4 °C, and the supernatants were collected. Lipid peroxidation end product malondialdehyde (MDA) was measured by a high-sensitivity thiobarbituric acid method using the MDA assay kit (S0131S, Beyotime, Shanghai, China). Protein concentration was measured by the BCA method and MDA results were normalized to the same protein load.

### 2.10. Determination of Glutathione (GSH)

The total GSH levels in cells and brain tissues were quantitatively measured using the GSH detection kit (S0052, Beyotime, Shanghai, China), and the experimental procedures strictly followed the steps outlined in the kit’s manual.

### 2.11. Propidium Iodide (PI) Staining

HT22 cells were incubated with 2 μM propidium iodide (PI, P4170, Sigma, St. Louis, MO, USA) at 37 °C for 30 min in the dark to prevent fluorescence quenching. Bright-field and red fluorescence images were then acquired using a fluorescence microscope (Nikon, Tokyo, Japan) to evaluate cell death based on membrane integrity loss.

### 2.12. Cell Viability Assays

Cell viability was measured using Cell Counting Kit-8(CCK-8) (C0037, Beyotime, Shanghai, China). HT22 cells were seeded at 5000 cells per well in a 96-well plate and treated appropriately, and 100 μL of 10% CCK-8 working solution was added to each well, gently mixed, and incubated at 37 °C for 90 min. Absorbance was measured at 450 nm using a microplate reader and cell viability was measured based on the survival rate.

### 2.13. Assay of Cellular Iron

After specific group processing, cells were collected via centrifugation and the intracellular ferrous ion (Fe^2+^) levels were quantitatively determined using an iron detection kit (E-BC-K881-M, Elabscience, Wuhan, China) according to the manufacturer’s instructions. The absorbance was detected at 593 nm using a microplate reader.

### 2.14. Western Blotting

Total protein was extracted from the peri-hematomal tissue and HT22 cells using RIPA lysis buffer with protease inhibitors (P0013B, Beyotime, Shanghai, China). Nuclear and cytoplasmic protein fractions were prepared using the Nuclear and Cytoplasmic Protein Extraction Kit (P0028, Beyotime, Shanghai, China) and protein concentration was measured using the BCA kit (Life-ilab, Shanghai, China). An amount of 20 μg of total protein per sample was separated by SDS-PAGE and transferred onto PVDF membranes. The membranes were blocked with 5% skim milk in TBST (Tris-buffered saline with 0.25% Tween-20) for 1 h at room temperature and then incubated with the following primary antibodies at 4 °C overnight: ACSL4 (1:5000, Proteintech, Rosemont, IL, USA, 22401-1-AP), GPX4 (1:1000, Proteintech, Rosemont, IL, USA, 30388-1-AP), Nrf2 (1:2000, Thermo Fisher, Waltham, MA, USA, PA5-27882), HO-1 (1:2000, Abcam, Cambridge, UK, ab189491), Histone H3 (1:2000, Proteintech, Rosemont, IL, USA, 17168-1-AP), and β-Actin (1:4000, Proteintech, Rosemont, IL, USA, 20536-1-AP). After washing, membranes were washed three times with TBST for 10 min each, incubated with appropriate horseradish peroxidase conjugated secondary antibodies (anti-mouse or anti-rabbit), diluted in 5% skim milk for 2 h at room temperature, and rinsed again three times with TBST for 10 min each. Protein bands were visualized using an enhanced chemiluminescence kit, and their intensities were measured by ImageJ software(ImageJ 1.x).

### 2.15. Reverse Transcription Quantitative Real-Time Polymerase Chain Reaction (RT-qPCR)

According to the reagent manual, total RNA was extracted from the tissue surrounding the hematoma and HT22 cells using Trizol reagent (Thermo, Waltham, MA, USA), and mRNA was reverse-transcribed into cDNA using a reverse transcriptase kit (R223-01, Vazyme, Nanjing, China). Real-time quantitative PCR was performed with SYBR qPCR Master Mix (Q312-02, Vazyme, Nanjing, China) on a Roche Lightcycler II, and was conducted using specific primers for the target gene and the reference gene β-Actin (Table 1). The cycle threshold (Ct) values for the target RNA were normalized to those of β-Actin, and the relative quantification of the data was analyzed using the 2^−ΔΔCT^ method (Livak).

### 2.16. Hematoxylin and Eosin (HE) Staining

Hematoxylin–eosin (HE) staining was conducted following standard procedures. Initially, the sections were washed three times in PBS buffer (Solarbio, Beijing, China), with each wash lasting 5 min. Subsequently, they were dehydrated in a series of ethanol solutions, and the sections were then stained in hematoxylin solution (Solarbio, Beijing, China) for 30 s, followed by incubation in a 1% hydrochloric acid solution for 5 s to achieve nuclear shrinkage. Afterward, the sections were rinsed in Scott’s tap water for 1 min to remove any residual hydrochloric acid and immediately stained with eosin solution for 30 s. Finally, the sections were dehydrated again through a graded ethanol series, and images were captured using an optical microscope.

### 2.17. Perls-DAB Staining

The iron content in brain tissue was detected by the Perls-DAB staining method. The brain slices were washed with PBS and then incubated in a mixture of potassium ferrocyanide and 2% hydrochloric acid of equal volume for 3 min. Subsequently, the slices were thoroughly rinsed with PBS. Then, the slices were incubated in a metal-enhanced DAB substrate solution (#34065, Thermo Fisher, Waltham, MA, USA) in the dark for 1 h to enhance the staining signal. Finally, the slices were dehydrated through a gradient of ethanol, cleared with xylene, and mounted for fixation. Images were then captured using an optical microscope.

### 2.18. Statistical Analysis

The statistics and plotting were performed using GraphPad Prism 10. Comparisons between two groups were made using t test, and comparisons between multiple groups were conducted with one-way ANOVA depending on the design of experiments. The significance was set to *p* < 0.05.

## 3. Results

### 3.1. BET Alleviates Hemin-Induced Neuronal Damage

**Figure 2:**
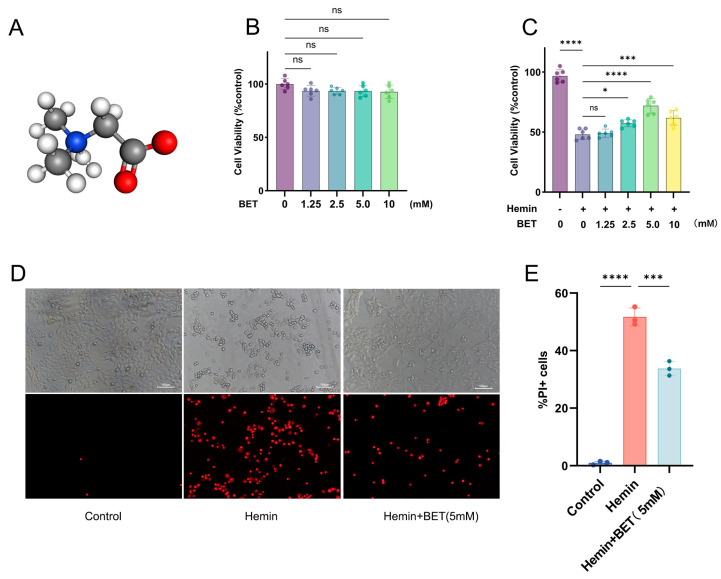
BET alleviates hemin-induced HT22 cell injury. Three-dimensional ball-and-stick model of BET, hydrogen atoms are colored white, carbon atoms gray, nitrogen atoms blue, and oxygen atoms red (**A**). The viability of HT22 cells treated with BET was detected by CCK-8 to evaluate the cytotoxicity of the drug (*n* = 6) (**B**). After pretreating HT22 cells with BET for 24 h, the cells were stimulated with hemin (20 μM), and the cell survival rate was detected (*n* = 6) (**C**). The effect of BET (5 mM) on the survival of HT22 cells was detected by PI staining (*n* = 3); scale bars: 100 μm (**D**). Count of PI-positive cells (*n* = 3) (**E**). Data are presented as the mean ± SD, ns: *p* > 0.05, * *p* < 0.05, *** *p* < 0.001, **** *p* < 0.0001.

We first determined the safe range of BET, with no significant cytotoxicity after 24 h of BET exposure to concentrations below 10 mM (Figure 2B), and then developed a cell injury model using 20 μM hemin [28,29]. HT22 cells were pretreated with BET 24 h before exposure to hemin for the best protective concentration. The CCK-8 assay demonstrated that hemin significantly reduced cell viability, whereas BET treatment effectively counteracted this effect in a dose-dependent manner, with 5 mM representing the optimal protective concentration (Figure 2C). PI staining showed that dead cells were significantly reduced with 5 mM BET (Figure 2D,E), so 5 mM was selected as the intervention concentration for subsequent experiments.

### 3.2. BET Alleviates Hemin-Induced Neuronal Ferroptosis and Oxidative Damage In Vitro

**Figure 3:**
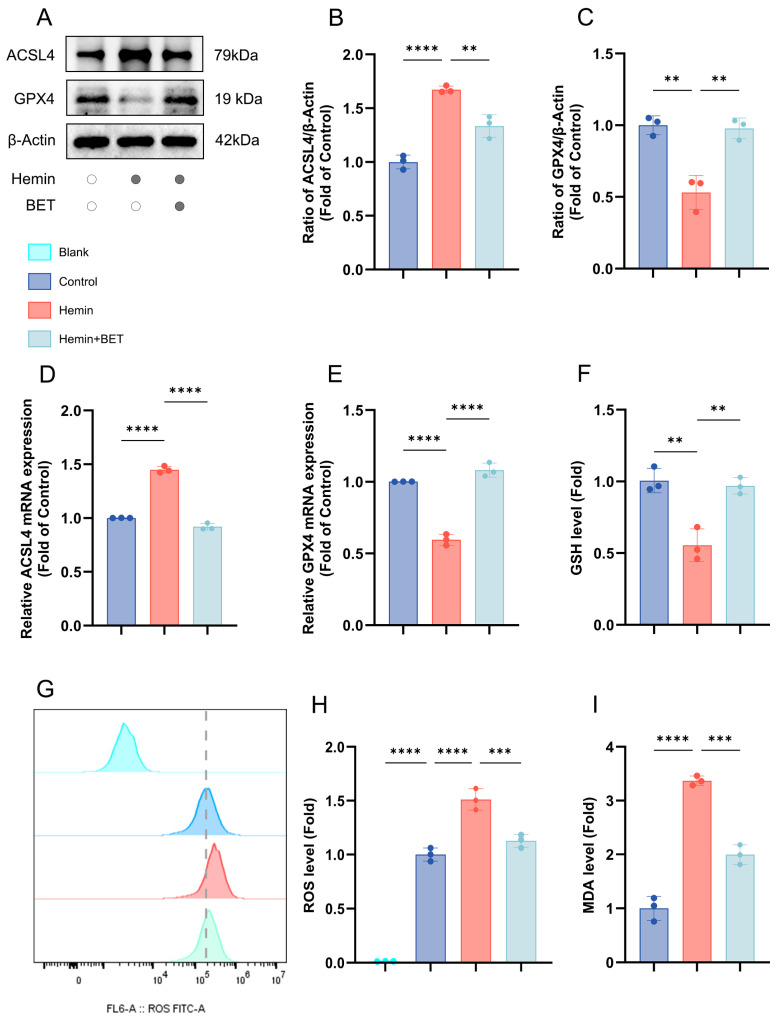
The effects of BET on the expression of ferroptosis-related molecules and oxidative stress indicators in an in vitro model of hemin-induced ICH. Representative Western blot bands for ACSL4 and GPX4 (*n* = 3) (**A**). Quantitative analysis of ACSL4 (**B**) and GPX4 (**C**) protein expressions (*n* = 3). The mRNA expressions of ACSL4 and GPX4 in the in vitro ICH model induced by hemin (*n* = 3), respectively (**D**,**E**). The relative expression levels of GSH (**F**), ROS (**G**,**H**), and MDA (**I**) in HT22 cells, dashed line: ROS level of the control group (*n* = 3). Data are presented as mean ± SD, ** *p* < 0.01, *** *p* < 0.001, **** *p* < 0.0001.

Western blot analysis revealed that hemin stimulation successfully induced ferroptosis, as evidenced by a significant down-regulation and up-regulation of GPX4 and ACSL4 protein expression, respectively, and BET pretreatment effectively reversed these changes in protein expression (Figure 3A–C). These results were further confirmed at the mRNA level through PCR experiments (Figure 3D,E). Under hemin stress, the level of the antioxidant and substrate for GPX4 reaction, GSH, decreased significantly, but BET treatment reversed the depleted GSH (Figure 3F). Flow cytometry analysis demonstrated that hemin induced ROS accumulation in HT22 cells, whereas subsequent treatment with BET markedly reduced intracellular ROS levels (Figure 3G,H). Iron-dependent lipid peroxidation is a key mechanism of ferroptosis and is closely related to secondary brain injury following ICH [30]. We evaluated MDA content (product of lipid peroxidation), finding that BET treatment reduced the rise in MDA due to hemin stimulation (Figure 3I). Thus, it is demonstrated that BET has an effective alleviating effect on lipid peroxidation during ferroptosis.

### 3.3. BET Reduces Hemin-Induced Neuronal Ferroptosis and Oxidative Damage Through the Nrf2/HO-1 Pathway

**Figure 4:**
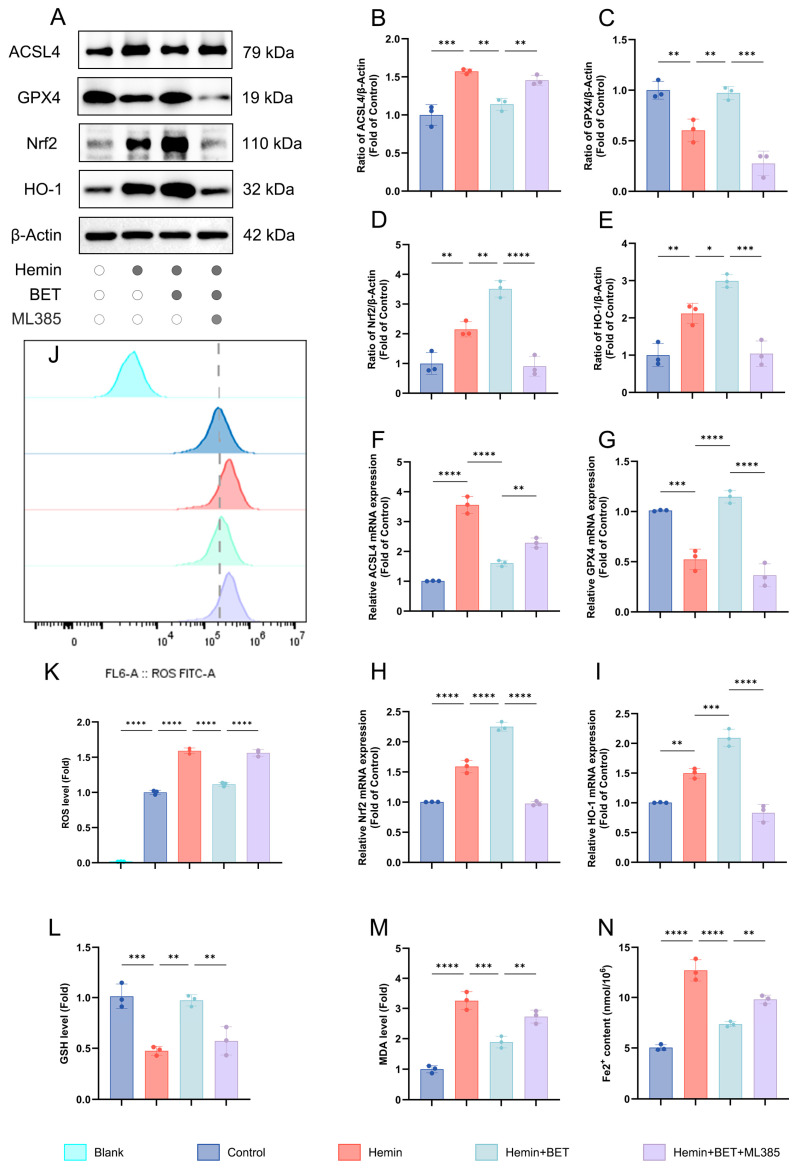
The effects of BET on ferroptosis markers, the Nrf2/HO-1 axis, and the oxidative stress response in a hemin-induced in vitro model of ICH. Representative Western blot bands for ACSL4, GPX4, Nrf2, and HO-1 (*n* = 3) (**A**). Quantitative analysis of ACSL4 (**B**), GPX4 (**C**), Nrf2 (**D**), and HO-1 (**E**) protein expressions (*n* = 3). The mRNA expressions of ACSL4, GPX4, Nrf2, and HO-1 in hemin-stimulated HT22 cells (*n* = 3), respectively (**F**–**I**). The relative expression levels of ROS (*n* = 3) (**J**,**K**), GSH (**L**), and MDA (**M**) in HT22 cells, dashed line: ROS level of the control group. The intracellular iron ion content (*n* = 3) (**N**). Data are presented as mean ± SD, * *p* < 0.05, ** *p* < 0.01, *** *p* < 0.001, **** *p* < 0.0001.

Based on previous studies, which have confirmed that BET can activate the Nrf2/HO-1 pathway to alleviate oxidative stress damage [19,21], we further explored the role and mechanism of this pathway in regulating key molecules of ferroptosis. Using the Nrf2 inhibitor ML385, we investigated the protective action of BET (5 mM) against hemin-induced neuronal ferroptosis. After hemin stimulation, the Nrf2/HO-1 pathway was activated with characteristic ferroptosis markers: decreased GPX4 and increased ACSL4. BET treatment increased Nrf2 and its downstream target HO-1 and reversed changes in GPX4 and ACSL4 (Figure 4A–E). This suggests that BET modulates the balance of ferroptosis molecules by activating the Nrf2/HO-1 pathway. At the transcriptional level, the PCR results were consistent with the protein data. Hemin stimulation increased Nrf2 and HO-1 mRNA levels, decreasing GPX4 and increasing ACSL4. BET treatment increased Nrf2 and HO-1 transcription and counteracted mRNA changes in GPX4 and ACSL4 (Figure 4F–I), which suggests that BET regulates ferroptosis via the Nrf2/HO-1 pathway at the transcriptional level. When the Nrf2-specific inhibitor ML385 was administered, not only was Nrf2/HO-1 expression suppressed at both the protein and mRNA levels, but the alterations in GPX4 and ACSL4 expression at both protein and gene levels were also reversed to an abnormal state comparable to that observed in the hemin group. BET decreased hemin-induced ROS production by activating Nrf2/HO-1, and treatment with ML385 reversed this effect and re-accumulated lipid ROS levels. BET suppressed MDA generation through this pathway and restored GSH levels (Figure 4J–M). In addition, the results of intracellular iron content detection showed that BET treatment could significantly alleviate the intracellular iron ion accumulation induced by hemin (Figure 4N). By contrast, treatment with ML385 reduced BET’s protective effects on the above markers (Figure 4J–M).

The aforementioned studies indicate that the Nrf2 signaling pathway plays a pivotal role in mediating the regulatory effects of BET on ferroptosis. Specifically, BET activates the Nrf2/HO-1 pathway, thereby modulating key ferroptosis-related molecules such as GPX4 and ACSL4, which contribute to its protective effect against hemin-induced neuronal ferroptosis. By contrast, ML385 exacerbates hemin-induced lipid peroxidation and ferroptosis, and attenuates the protective effects of BET in this context.

### 3.4. BET Promotes Nuclear Translocation of Nrf2 In Vitro

**Figure 5:**
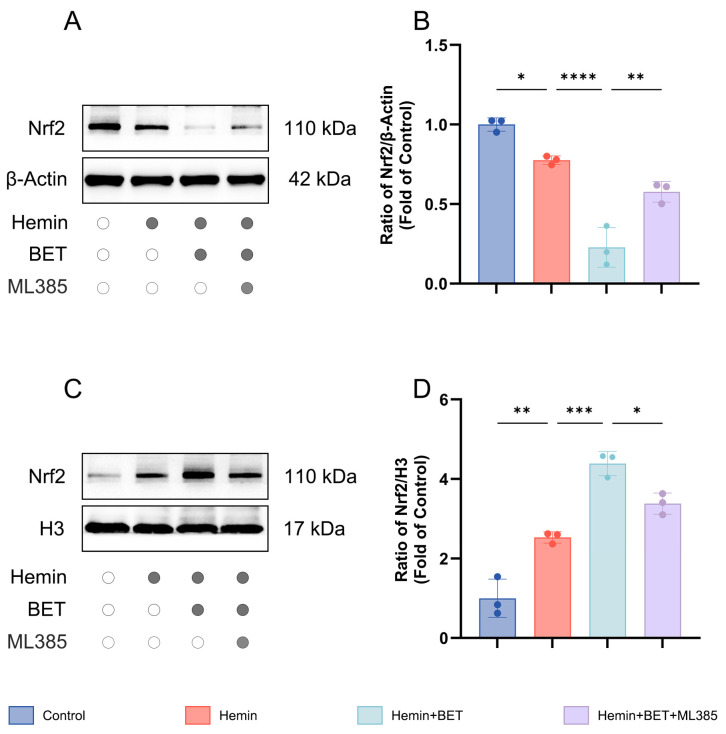
Subcellular localization of Nrf2 in hemin-stimulated HT22 cells with BET intervention. Representative Western blot images showing cytoplasmic Nrf2 expression (*n* = 3) (**A**). Analysis of the expression level of Nrf2 in the cytoplasm (*n* = 3) (**B**). Representative Western blot images showing nuclear Nrf2 expression (*n* = 3) (**C**). Analysis of the expression level of Nrf2 in the nucleus (*n* = 3) (**D**). Data are presented as mean ± SD, * *p* < 0.05, ** *p* < 0.01, *** *p* < 0.001, **** *p* < 0.0001.

Nrf2 must be transferred into the cell nucleus to fully exert its transcriptional regulatory function [31]. To assess whether BET affects the nuclear translocation of Nrf2, we analyzed Nrf2 distribution in nuclear fractions and in cytoplasmic fractions by Western blot. The results showed that Nrf2 was distributed in the nucleus and cytoplasmic fractions, and hemin stimulation promoted Nrf2 nuclear translocation (and reaches a higher nuclear level than the control does). BET treatment further enhanced Nrf2 nuclear accumulation (indicating it actively facilitates Nrf2 nuclear import), and cytoplasmic Nrf2 levels dropped. The Nrf2 inhibitor ML385 significantly suppressed BET nuclear accumulation (Figure 5A–D). Thus, we conclude that BET activates the Nrf2/HO-1 pathway and enhances Nrf2 transcriptional activity by promoting its nuclear translocation.

### 3.5. BET Dose-Dependently Improves Neurological Deficits in ICH Mice In Vivo

**Figure 6:**
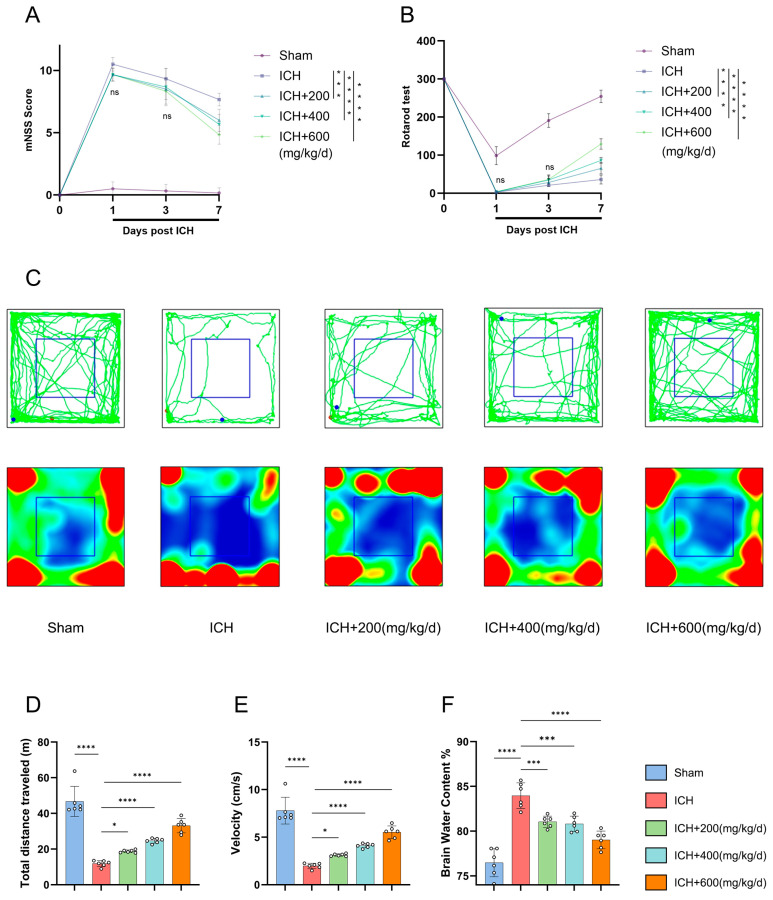
Assessment of neurological function and brain edema severity in mice after ICH. Modified neurological severity scores (mNSSs) on days 1, 3, and 7 after ICH (*n* = 6) (**A**). Rotarod test data on days 1, 3, and 7 after ICH (*n* = 6) (**B**). Movement trajectories and heatmaps of mice in the open field test at 7 days post ICH, green lines indicate movement trajectories; red and blue dots represent movement start and end points, respectively; blue boxes denote the central area. red areas in heatmaps indicate frequent visits by mice, while blue areas indicate rare visits (*n* = 6) (**C**). Distance traveled and average speed on day 7 after ICH (*n* = 6) (**D**,**E**). Brain water content on day 7 after ICH (*n* = 6) (**F**). Data are presented as mean ± SD, ns: *p* > 0.05, * *p* < 0.05, *** *p* < 0.001, **** *p* < 0.0001.

An ICH model was established in mice by stereotactic injection of collagenase [24]. Based on published research in the relevant field, we designed three concentration gradient BET intervention schemes [25]. Neurological function measured by the mNSS was severely impaired in all mice with ICH on day 1 post ICH, and the therapeutic effect of BET was time-dependent. While no significant differences between the BET groups and ICH group were observed in the early stage (days 1 and 3), all BET-treated groups showed dose-dependent neurological improvement by day 7 (Figure 6A). Motor coordination was assessed using the rotarod test. ICH significantly reduced the latency to fall as opposed to the sham group. Although no differences were detected in the groups initially, all BET treatment groups, showed significantly longer latencies by day 7 (Figure 6B). To assess motor function, we performed an open-field test on day 7. ICH significantly reduced spontaneous locomotor activity by significant reductions in total distance traveled and average speed, while BET treatment reversed these deficits, and the high-dose group restored activity to near-normal levels (Figure 6C–E). We observed that the water content of brain tissue in the ICH group significantly increased, indicating the formation of brain tissues edema. Different doses of BET treatment could effectively alleviate this pathological change, and the degree of improvement was positively correlated with the drug concentration. Among them, the high-dose group showed the best neuroprotective effect in alleviating brain edema (Figure 6F).

In this study, we identified continuous intraperitoneal administration of high-dose BET (600 mg/kg/day) over 7 days as the optimal therapeutic regimen. This regimen demonstrated significant efficacy in ameliorating neurological deficits, promoting motor function recovery, and alleviating cerebral edema. Consequently, it was selected for subsequent in-depth mechanistic investigations.

### 3.6. BET Alleviates Ferroptosis and Oxidative Damage in ICH-Induced Mice by Modulating the Nrf2/HO-1 Signaling Pathway

**Figure 7:**
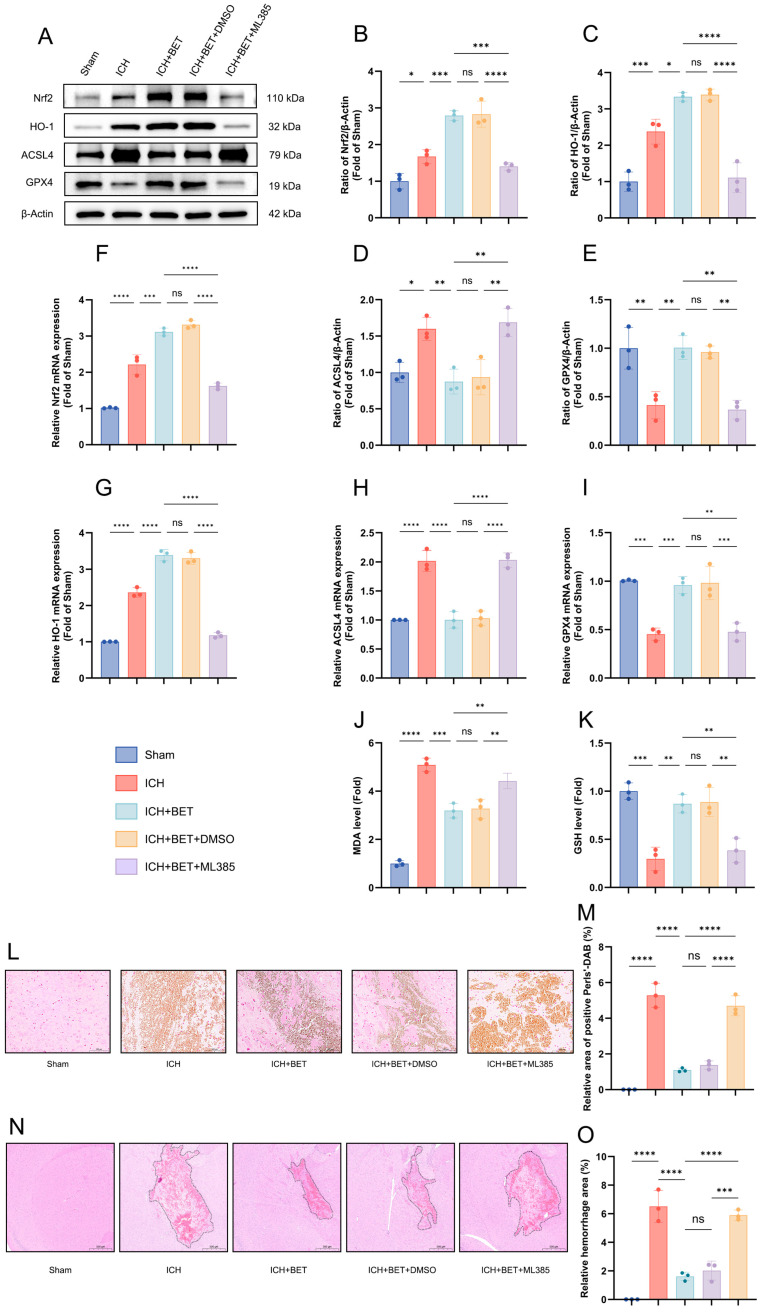
Ferroptosis-related biomarkers, oxidative stress levels, and histopathological changes in mice brain tissues 7 days after ICH. Representative Western blot images of ACSL4, GPX4, Nrf2, and HO-1 in mice brain tissues on the 7th day after ICH (*n* = 3) (**A**). Quantitative analysis of protein expression of ACSL4, GPX4, Nrf2, and HO-1 (*n* = 3) (**B**–**E**). mRNA expression levels of ACSL4, GPX4, Nrf2, and HO-1 in mice brain tissues on day 7 after ICH (*n* = 3) (**F**–**I**). The content of GSH and MDA in brain tissues on the 7th day after ICH (*n* = 3) (**J**,**K**). Perls-DAB staining results in brain tissues; scale bars: 100 μm (*n* = 3) (**L**). The proportion of Perls-DAB staining-positive areas (**M**). HE staining of brain tissues, with the dashed line delineating the hematoma area; scale bars: 500 μm (*n* = 3) (**N**). The ratio of the hematoma area (*n* = 3) (**O**). Data are presented as mean ± SD, ns: *p* > 0.05, * *p* < 0.05, ** *p* < 0.01, *** *p* < 0.001, **** *p* < 0.0001.

To determine whether BET regulates ferroptosis-related molecules via Nrf2 in vivo, we studied the expression of Nrf2, HO-1, and ferroptosis markers (ACSL4 and GPX4) using Western blot and PCR. Compared with the sham group, the ICH group exhibited significant upregulation of both Nrf2 and its downstream target gene HO-1 at the protein and mRNA levels, indicating activation of the endogenous antioxidant pathway. Furthermore, BET treatment further increased the expression of Nrf2 and HO-1. This effect was abolished by ML385, indicating that BET action depends on the activation of the Nrf2/HO-1 pathway (Figure 7A–C,F,G). ICH induces a significant increase in ACSL4 and a decrease in GPX4, which indicates ferroptosis activation, and BET treatment successfully reversed the changes (Figure 7D,E,H,I). Co-treatment with ML385 abolished this protective effect. Moreover, BET suppressed the increase in MDA and restored GSH levels (Figure 7J,K), and promoted antioxidant effects, further proof that the Nrf2 pathway regulates oxidative balance (Figure 7J,K). Histological results revealed significant iron deposition and hematoma in ICH mice. BET treatment reduced iron deposition and hematoma size in brain tissue, but ML385 treatment partially reversed this effect (Figure 7L–O).

Our findings demonstrate that BET exerts comprehensive neuroprotection in ICH by activating the Nrf2/HO-1 pathway. This activation enhances antioxidant capacity, reduces iron deposition and lipid peroxidation, inhibits ferroptosis, and promotes hematoma resolution.

### 3.7. BET Promotes Nuclear Translocation of Nrf2 In Vivo

**Figure 8:**
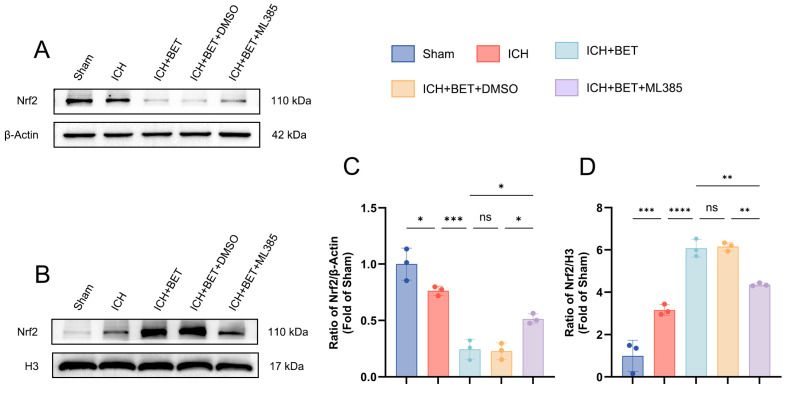
Characteristics of cytoplasmic-nuclear distribution of Nrf2 in mice brain tissues on day 7 post ICH. Representative Western blot images of cytoplasmic and nuclear Nrf2 in mice brain tissues on the 7th day after ICH (*n* = 3) (**A**,**B**). Quantitative analysis of cytoplasmic and nuclear Nrf2 protein levels in mice brain tissues on day 7 post ICH (*n* = 3) (**C**,**D**). Data are presented as mean ± SD, ns: *p* > 0.05, * *p* < 0.05, ** *p* < 0.01, *** *p* < 0.001, **** *p* < 0.0001.

We investigated the effect of BET on Nrf2 localization in vivo. Compared with the sham group, ICH alone reduced cytoplasmic Nrf2 levels and increased its nuclear concentrations, and this shift was enhanced by BET treatment. ML385 inhibited the nuclear accumulation of Nrf2 promoted by BET (Figure 8A–D). Together, our results show that BET significantly promotes the nuclear translocation of Nrf2 in vivo.

## 4. Discussion

In our study, we explored BET’s effects on ferroptosis and oxidative stress after ICH in vitro and in vivo. In vitro, BET attenuated hemin-induced HT22 cell death and inhibited ferroptosis by promoting Nrf2 nuclear translocation, upregulating HO-1, reducing ROS, increasing GSH, upregulating GPX4, and decreasing ACSL4 and MDA. In vivo, BET ameliorated motor deficits and brain edema dose-dependently; high-dose BET suppressed ferroptosis, reduced brain iron deposition, and accelerated hematoma clearance. Nrf2 pathway inhibition abolished BET’s protective effects and restored ferroptosis.

Secondary brain injury following an ICH is one of the key factors in poor patient outcomes due to a pathologic cascade initiated by erythrocyte lysis and hemoglobin degradation [32]. The breakdown of hemoglobin by hemin oxygenase-1 (HO-1) releases iron [33,34], and iron overload causes the Fenton reaction, excessive ROS, ferroptosis, and oxidative damage [35,36,37]. Therefore, targeted intervention in ferroptosis—through strategies such as regulation of iron ion metabolism, inhibition of lipid peroxidation, and upregulation of GPX4 expression—has emerged as a promising therapeutic approach for mitigating secondary brain injury following an ICH and improving long-term patient outcomes, holding significant potential for advancing the optimization of clinical treatment strategies [38,39,40].

Our results support this approach, showing that BET is twofold-protected. First, BET increases resistance to ferroptosis by decreasing ROS levels and upregulating the main antioxidant enzyme GPX4. Second, BET decreases the burden of peroxidation at the source by suppressing ACSL4, the enzyme responsible for pro-ferroptotic metabolism. These findings indicate that BET is a promising therapeutic candidate for ICH.

Nrf2 is the main regulator of the antioxidant response [41,42]. Under oxidative stress conditions, such as ICH, Nrf2 can be induced to activate transcription of cytoprotective genes (HO-1 and GPX4), thus decreasing intracellular lipid peroxide accumulation. Previous studies have demonstrated that moderate targeted regulation of Nrf2 can suppress ferroptosis and oxidative stress levels, enhancing the brain’s resilience to stress and pressure [43,44,45,46,47]. Li et al. used BSA nanoparticles to activate the Nrf2 pathway, which enhanced GPX4 and reduced ICH injury [48]. Zhou et al. showed that WFA treatment reduced ferroptosis and oxidative stress in ICH via the Nrf2/HO-1 pathway [49]. Therefore, targeting Nrf2 regulation represents a pivotal strategy for suppressing ferroptosis following an ICH.

BET, as a stable and non-toxic substance widely present in animals, plants, and microorganisms, can pass through the blood–brain barrier and be transported to the brain via the betaine-GABA transporter protein (BGT-1) [13,50]. BET has a wide range of functions in anti-inflammation, antioxidation, and regulation of mitochondrial function [15,17]. In the nervous system, for example, BET inhibits microglial pyroptosis by suppressing the NLRP3/caspase-1/GSDMD pathway in an m6A-YTHDF2-dependent manner, thereby improving cognitive impairment [51]. The research by Hui et al. shows that BET plays an important role in alleviating depressive-like behaviors and cognitive impairments caused by METH abuse, and has potential applications in preventing and treating substance addiction [52]. Previous studies have focused on BET in neurodegenerative diseases and mental disorders, mainly related to cellular death mechanisms such as antioxidant stress and anti-apoptosis. Excessive accumulation of ROS after ICH leads to oxidative stress, which is the core driving factor of ferroptosis [53,54].

Although some studies have suggested that BET activates Nrf2 for anti-inflammatory and antioxidant effects [19,20,21], ferroptosis inhibition via the Nrf2/HO-1 pathway following an ICH has not been investigated. Moreover, previous research results have shown that the Nrf2/HO-1 pathway is a key pathway for exerting antioxidant stress and anti-ferroptosis effects [49,55], and in [19,20,21], BET has been confirmed to exert antioxidant stress and anti-inflammatory effects by activating Nrf2 expression; however, whether it can exert anti-ferroptosis effects through activating the Nrf2/HO-1 pathway after ICH has not been explored. Our work fills this gap. Our study has demonstrated the positive role of BET in resisting iron-induced cell death, providing more possibilities for the future treatment of ICH.

However, this study has certain limitations warranting further investigation in subsequent work. First, we demonstrated that BET exerts anti-ferroptotic effects via the Nrf2/HO-1 pathway in ICH. Studies have shown that BET can exert its function by inhibiting pyroptosis [51]. Whether BET coordinately modulates pyroptosis, apoptosis, and other programmed cell death forms via this pathway merits further exploration. More experimental evidence is required to clarify whether BET can mediate its neuroprotective effects through other potential signaling cascades. Furthermore, the extent of Nrf2 activation awaits further in-depth investigation in future studies. Notably, no clear side effects were observed in both in vivo and in vitro experiments at the doses administered in the present study. Second, we only investigated BET’s regulatory role in ICH via ferroptosis-related molecular expression. The regulatory effects of BET on characteristic morphological hallmarks of ferroptosis (e.g., mitochondrial morphological alterations) will be incorporated into our follow-up research framework, with systematic validation using transmission electron microscopy. Notably, BET has FDA approval for homocystinuria treatment at a recommended dose of 6–9 g/d [56,57]. Its feasibility, optimal dosing strategy, and safety profile for translational application in ICH patients need further establishment through preclinical and clinical studies.

## 5. Conclusions

**Figure 9:**
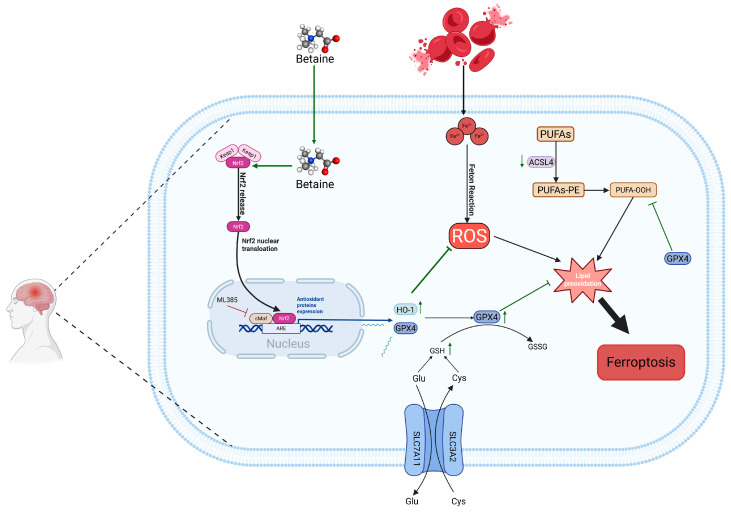
Mechanism diagram of this study. BET, betaine; Nrf2, nuclear factor E2-related factor 2; HO-1, heme oxygenase 1; ACSL4, long-chain acyl-CoA synthetase 4; GPX4, glutathione peroxidase 4; ROS, reactive oxygen species; PUFAs, polyunsaturated fatty acids; SLC3A2, solute carrier family 3 member 2; SLC7A11, solute carrier family 7 member 11; arrows reflect the upstream-downstream regulatory interplay of signaling pathways and the up-regulation or down-regulation trends of molecular expression; green arrows represent BET’s effective regulatory role; arrows with a transverse bar denote targeted inhibition.

In summary, BET can effectively activate the Nrf2/HO-1 signaling pathway by promoting the nuclear translocation of Nrf2, thereby inhibiting the generation of ROS, downregulating the expression of ACSL4, and upregulating the levels of GPX4 and GSH. This significantly reduces lipid peroxidation reactions and ultimately exerts inhibitory effects on ferroptosis and oxidative stress after ICH (Figure 9). Additionally, BET can improve neurological deficits after ICH, promote hematoma clearance, alleviate cerebral edema, and, with its good biocompatibility and non-toxic properties, and provide a potential new intervention strategy for the treatment of ICH.

## Figures and Tables

**Table 1:** Primer sequences. Nfe2l2, nuclear factor E2-related factor 2; Hmox1, heme oxygenase 1; Acsl4, long-chain acyl-CoA synthetase 4; Gpx4, glutathione peroxidase 4; Actb, beta-actin.

Gene	RefSeq-ID	Primer Sequence (5′-3′)	
*Nfe2l2*	NM_010902.5	F	ATAGCTGAGCCCAGTATC
		R	CATGCACGTGAGTGCTCT
*Hmox1*	NM_010442.2	F	AAGACTGCGTTCCTGCTCAAC
		R	AAAGCCCTACAGCAACTGTCG
*Acsl4*	NM_207625.2	F	GCTATCTCCTCAGACACACCGA
		R	AGGTGCTCCAACTCTGCCAGTA
*Gpx4*	NM_008162.4	F	ACAAGAACGGCTGCGTGGTGAA
		R	GCCACACACTTGTGGAGCTAGA
*Actb*	NM_007393.5	F	CATTGCTGACAGGATGCAGAAGG
		R	TGCTGGAAGGTGGACAGTGAGG

## Data Availability

The original contributions presented in this study are included in the article. Further inquiries can be directed to the corresponding authors.

## Abbreviations

The following abbreviations are used in this manuscript:

ICH	Intracerebral hemorrhage
BET	Betaine
Nrf2	Nuclear factor E2-related factor 2
HO-1	Heme oxygenase 1
ACSL4	Long-chain acyl-CoA synthetase 4
GPX4	Glutathione peroxidase 4
MDA	Malondialdehyde
GSH	Glutathione
ROS	Reactive oxygen species
PUFAs	Polyunsaturated fatty acids
PLOOHs	Phospholipid hydroperoxides
GCL	Glutamate-cysteine ligase
GS	Glutathione synthetase
